# Alkaloids solenopsins from fire ants display *in vitro* and *in vivo* activity against the yeast *Candida auris*

**DOI:** 10.1080/21505594.2024.2413329

**Published:** 2024-10-07

**Authors:** Leandro Honorato, Jhon Jhamilton Artunduaga Bonilla, Larissa Ribeiro da Silva, Julio Kornetz, Daniel Zamith-Miranda, Alessandro F. Valdez, Joshua D. Nosanchuk, Eduardo Gonçalves Paterson Fox, Leonardo Nimrichter

**Affiliations:** a Universidade Federal do Rio de Janeiro, Instituto de Microbiologia Paulo de Góes, Departamento de Microbiologia Geral, Rio de Janeiro, Brazil; b Departments of Medicine (Division of Infectious Diseases) and Microbiology and Immunology, Albert Einstein College of Medicine, Bronx, New York, USA; c Programa de Pós-Graduação em Ambiente e Sociedade (PPGAS), Universidade Estadual de Goiás (UEG), Quirinópolis, Brazil; d Rede Micologia, RJ, FAPERJ, Rio de Janeiro, Brazil

**Keywords:** *Candida auris*, *Solenopsis invicta*, solenopsins, alkaloid, biofilm, candidiasis

## Abstract

The urgency surrounding *Candida auris* as a public health threat is highlighted by both the Center for Disease Control (CDC) and World Health Organization (WHO) that categorized this species as a priority fungal pathogen. Given the current limitations of antifungal therapy for *C. auris*, particularly due to its multiple resistance to the current antifungals, the identification of new drugs is of paramount importance. Some alkaloids abundant in the venom of the red invasive fire ant (*Solenopsis invicta*), known as solenopsins, have garnered attention as potent inhibitors of bacterial biofilms, and there are no studies demonstrating such effects against fungal pathogens. Thus, we herein investigated the antibiotic efficacy of solenopsin alkaloids against *C. auris* biofilms and planktonic cells. Both natural and synthetic solenopsins inhibited the growth of *C. auris* strains from different clades, including fluconazole and amphotericin B-resistant isolates. Such alkaloids also inhibited matrix deposition and altered cellular metabolic activity of *C. auris* in biofilm conditions. Mechanistically, the alkaloids compromised membrane integrity as measured by propidium iodide uptake in exposed planktonic cells. Additionally, combining the alkaloids with AMB yielded an additive antifungal effect, even against AMB-resistant strains. Finally, both extracted solenopsins and the synthetic analogues demonstrated protective effect *in vivo* against *C. auris* infection in the invertebrate model *Galleria mellonella*. These findings underscore the potent antifungal activities of solenopsins against *C. auris* and suggest their inclusion in future drug development. Furthermore, exploring derivatives of solenopsins could reveal novel compounds with therapeutic promise.

## Introduction

The opportunistic yeast *Candida auris* is a worldwide threat, with clinical cases and outbreaks reported across the globe [1–8]. Epidemiological studies have identified six major clades underlying *C. auris* genetic diversity, distributed across distinct geographical regions [9–11]: Clade-I is the South Asian clade, clade-II is the East Asian clade, clade-III is the South African clade, clade-IV clusters in Colombian, Venezuelan and other South American countries, clade-V is in Iran [9,11] and clade VI at the Indomalayan zone, with a close relationship to clade IV [12]. However, recent studies showed that *C. auris* clades are not geographically restricted to their original locations, confirming that *C. auris* has a high capacity to circulate [13].

Remarkably, most isolates from *C. auris* are multi-drug resistant [14,15]. The primary class of drugs used to combat candidiasis caused by *C. auris* are the echinocandins, with amphotericin B being recommended for cases where patients are clinically unresponsive or develop chronic fungemia lasting more than 5 days [16,17]. To emphasize on the emergency of *C. auris* resistant strains, a study published by Kilburn and colleagues [18] revealed that 99.8% of the clinical isolates in New Jersey and New York from 2016 to 2020 were resistant to fluconazole (FLU) and 50% resistant to amphotericin B (AMB). In addition, 4% of the New York strains were resistant to echinocandins, and 1% was pan-resistant. Biofilm formation, characterized by the aggregation of microorganisms on surfaces and the secretion of an extracellular matrix, has emerged as a significant factor contributing to the development of microorganisms resistance, including *C. auris* species [15,19,20]. Furthermore, *C. auris* is a persistent skin colonizer and can be transmitted from patients to healthcare workers, initiating a chain of transmission to other patients [21,22]. In healthcare settings, outbreaks of *C. auris* are well documented with an overall mortality rate of 39% [23].

It is thus imperative that new alternative treatments be provided to control colonizer persistence, transmission, and mortality caused by *C. auris*. In this context, the natural products known as alkaloids have been only partially exploited. Alkaloids are a heterogenous assembly of cyclic organic amines primarily found in plants as secondary metabolites [24]. They exhibit a wide range of effects, including anti-inflammatory, analgesic, stimulant, psychotropic, among others, so that some alkaloids have been exploited extensively by pharmaceuticals, cosmetic and food industries [25–27]. Furthermore, some alkaloids are notably toxic to pathogens, even at low doses, a property that has been addressed with antimicrobial properties [28–30]. For instance, Carvalho and colleagues have demonstrated that the animal piperidine alkaloids known as “solenopsins” suppress biofilm formation by the pathogenic bacterium *Pseudomonas fluorescens* [31].

Solenopsins are abundant in the venoms of invasive ants known as “imported fire ants” [32]. Structurally, solenopsins are asymmetrical di-substituted derivatives of a piperidine ring, displaying an odd-numbered hydrocarbon sidechain varying in length from 9 to 17 carbons [33]. Their analogues include optical and geometrical isomers and unsaturated derivatives, and their relative abundance in fire ant venoms is primarily species-specific and secondarily caste-specific [34,35]. Apart from delivering several physiological effects, these alkaloids are well-known as antimicrobials [36–38]. Alkaloids can kill diverse Gram positive and negative pathogenic bacteria – such as *Staphylococcus aureus*, *Streptococcus pneumoniae, Staphylococcus aureus, Enterococcus faecalis*, and *Escherichia coli* [39]. Moreover, they are also effective as fungicides against numerous pathogens of agricultural importance, such as *as Botrytis cinerea, Fusarium oxysporum, Phytophthora nicotianae, Beauveria bassiana, Metarhizium anisopliae*, and *Paecilomyces fumosoroseus* [40–42]. However, the efficacy of these alkaloids against human fungal pathogens has not been investigated.

In this study, we investigated the antifungal properties of solenopsins against different strains of the pathogenic yeast *C. auris*. We further investigated a potential synergistic effect of these alkaloids combined with AMB or FLU and exploited their ability to inhibit biofilm formation by *C. auris*. Finally, we tested the effect of these alkaloids in a candidiasis *in vivo* model using *Galleria mellonella* caterpillars. Taken together, our data suggest that solenopsins could be a potential strategy to control *C. auris*.

## Materials and methods

### Fungal strains and culture conditions

*C. auris* CDC strains 381 (clade II), 382 (clade I), 383 (clade III), 384 (clade III), 385 (clade IV) and 390 (clade I) were maintained in Sabouraud Dextrose Agar (SDA). The respective clade for each *C. auris* strain is shown in https://wwwn.cdc.gov/ARIsolateBank/Panel/PanelDetail?ID=2. Aggregative (CDC 382, 383, 384, 385) and non-aggregative (single-celled) (CDC 381 and 390) phenotype strains were tested (D. Zamith-Miranda, personal communication, May 2023). To perform experiments, each strain was cultivated in Sabouraud broth (2% glucose and 1% peptone) at 30 °C under agitation (150 RPM) for 24 h. Yeasts were washed with phosphate buffer saline – PBS (137 mm NaCl, 2.7 mm KCl, 1.5 mm KH_2_PO_4_, 8.1 mm Na_2_HPO_4_, pH 7.4) 3 times, enumerated using a hemocytometer and transferred to RPMI 1640 (Gibco – Brazil) buffered to pH 7.0 with 0.165 M morpholinopropanesulfonic acid (MOPS, USA) for the experiments.

### Alkaloid purification and synthetic alkaloids

A purified extract containing only solenopsins (henceforth “natural mixture,” NM) was obtained from workers of the red imported fire ant *Solenopsis invicta* Buren (i.e. any winged individuals were discarded). An active nest was located at an urban city park at the city of Guangzhou, R.P. China, and collected into a plastic bucket using an ordinary shovel. The nest was brought to the laboratory and separated from the soil by slow flooding [43] and the obtained ants were transferred alive into a plastic tray to allow them to clean themselves (estimated 19 g of workers). The ants were then cold-anesthetized in a common fridge and dropped into a mixture of water and hexane (1:5) for alkaloid extraction as described in Fox et al. [44]. The obtained organic extract (i.e. ca. 150 mL hexane containing fire ant alkaloids and hydrocarbons) was concentrated by evaporation to 1 mL and carefully applied into a 2 mm (internal diameter) 43 cm-long glass column loaded with a slur of 20 g 300–400 mesh silica in pure hexane for proceeding for normal phase chromatography as described in Shi et al. [35], with modifications. In short, the extract was washed with an increasing hexane:acetone gradient up to the concentration of 14:6 at the total elution volume 110 mL, with a final elution in 20 ml of acetone:TEA 95:5, delivering a purified extract containing cis-/trans-isomers of analogues of the piperidine alkaloids known as solenopsins. Approximately 18 mg of concentrated alkaloids were obtained from the whole-nest extraction protocol, representing a yield of 0.1% (w/w) from the original mass of ants. The alkaloids extract was checked for purity by injection into a gas chromatograph system [45]. Briefly, after solvent evaporation, the relative proportions of solenopsin analogues was determined by gas chromatography-mass spectrometry (GC – MS) coupled with a Shimadzu GCMS-QP2010 plus system equipped with a RTX-5 MS capillary column of fused silica (measuring 30 m long, internal diameter = 0.25 mm) (Restek, Bellefonte, PA, USA). Equipment settings for chromatography were as in Fox et al. [46]. The mass spectra were obtained through electron impact (EI) at 70 eV; solenopsins were identified based on comparison with their published GC-MS spectra [47,48]. Relative quantitation of peaks was determined by taking the ratios of the total peak area of each compound in the sample.

A synthetic mixture of solenopsins (henceforth “synthetic mixture,” SM) was prepared in high purity hexane, mimicking the recorded relative proportions in the natural extract. As previously described [49], synthetic racemic solenopsins were purchased from WuXi AppTec (ShangHai, China) after synthesis, following Pianaro et al. for cis- isomers and Herath and Nanayakkara for trans- isomers [50,51]. The SM sample mimicking the natural venom extract was obtained by synthetic analogues of the main components (i.e. those present at >10% of total peak area in the natural extract) in the analytical-grade hexane grade using precision Hamilton syringes at volumes following their relative proportions observed in the *S. invicta* workers extract described above. Once the synthetic mixture approached similar approximate proportions according to consecutive GC-MS chromatograms, the hexane solvent vehicle was completely evaporated under a nitrogen flow, leaving a clear oily mixture. Stock solutions for NM and SM were prepared in ethanol 100% for the described bioassays.

### In vitro antifungal tests

Antifungal tests were carried out according to the Clinical and Laboratory Standards Institute (CLSI) in 96-well microtiter plates based on the document M27-A3 [52]. Yeasts were prepared for a final suspension of 2.5 × 10^3^/mL in RPMI and the alkaloid concentration (NM and SM) ranged from 0.04 to and 22.4 µg/mL. Microplates were incubated at 35 °C for 48 h. As controls, untreated yeasts were incubated with either AMB at concentrations of 0.007 to 4 µg/mL or FLU at 1 to 512 µg/mL. Growth was determined visually and by spectrophotometric (λ: 540 nm) measurements after 24 and 48 h.

### Drug combination

Antifungal interactions between NM (0.04 to 22.4 µg/mL) and AMB (0.25 to 16 µg/mL) were evaluated using the checkerboard dilution assay [53]. Using 96-well plates, the alkaloids and the individual drug were serially diluted in a final volume of 100 µL of RPMI. Then, 100 µL of 5 × 10^3^ yeasts/mL were added to each well and incubated at 35°C for 48 h. The optical density was measured at 540 nm using a microplate reader (Biotek ELx808). The FICi was calculated as the total of ratios between the combined MIC and the individual MIC of each drug in each pair as follows: FICi = (MIC combined/MIC drug A alone) + (MIC combined/MIC drug B alone). The combinatory effect is considered synergistic whenever the FICi was ˂0.5, indifferent whenever the FICi is 0.5–4.0, and antagonistic whenever the FICi is >4.0 [53,54]. The drug interactions were also analyzed by the Bliss independence model using the SynergyFinder web application [55]. A score less than −10 is considered antagonistic interaction; from −10 to 10 additive interaction, and larger than 10 the interaction between two drugs is considered synergistic.

### Biofilm formation

Biofilms were developed following the previously established protocol described in [56]. Yeast cells were initially cultivated in Sabouraud broth for 24 h at 30°C under agitation (150 rpm). Cells were then washed three times with PBS and the suspension adjusted to 10^7^ cells/mL in RPMI. A final volume of 100 μL per well was transferred into a flat-bottom 96-well polystyrene microtiter plate and incubated for 90 min without agitation at 37°C. Non-adhered yeasts were removed by washing with PBS and the wells filled with 200 μL of RPMI in the presence of NM or SM at different concentrations (10, 25, 50, or 100 μg/mL) and the plates incubated for 48 h under the same conditions for biofilm formation experiments. For experiments with mature biofilm, cells were incubated with 200 μL of RPMI for 24 h at 37°C. After incubation, cells were washed with PBS three times and incubated 200 μL of RPMI in the presence of NM or SM at different concentrations (10, 25, 50, or 100 μg/mL) and the plates incubated for 24 h under the same conditions. Plate wells containing only RPMI were used to zero the reader as blanks. Controls were performed in the presence of RPMI alone or supplemented with 1% ethanol in PBS (i.e. the same concentration used for the alkaloids). The supernatant of each well was removed by pipetting and, subsequently, the wells were washed three times with PBS to remove any nonadherent cells.

### Biofilm parameters (metabolic activity and biomass)

The metabolic activity of the biofilm was determined using a colorimetric assay to measure the metabolic reduction of 2,3-bis (2-methoxy-4-nitro-5-sulfophenyl)-5-[(phenylamino) carbonyl]-2 h-tetrazolium hydroxide (XTT; Sigma – USA) to a water-soluble brown formazan product [57]. The XTT/menadione solution was prepared by dissolving 2 mg XTT in 10 mL of pre-warmed PBS, which was supplemented with 100 μL of a stock solution of menadione (0.4 mm in acetone). The XTT/menadione solution (200 μL) was added to the plate wells and incubated at 37°C for 3 h in the dark. Color development in the supernatant was quantified using a microplate reader at 490 nm (Biotek ELx808™). Biomass quantification was developed using crystal violet or safranin staining [57,58]. Firstly, methanol (Sigma – USA) was used to fix the biofilms for 15 min at room temperature. The supernatant was then discarded, and the plates were air-dried for 5 min. Afterwards, 0.4% crystal violet (Sigma – USA) was added to the wells, and the plates were incubated for 20 min at room temperature. The wells were washed once with PBS to remove the excess stain, and the bound dye was then eluted with 33% acetic acid (Sigma – USA) for 5 min. The solution was transferred to a new 96-well plate, and the absorbance was measured using a microplate reader at 540 nm (Biotek ELx808™). For safranin staining, 0.1% safranin (Sigma – USA) diluted in PBS was incubated with nonfixed biofilms at room temperature for 5 min. Afterwards, the wells were washed once with PBS, and the bound dye was eluted with 30% acetic acid. The eluate was transferred to a new 96-well plate, and the absorbance was quantified using a microplate reader at 540 nm (Biotek ELx808™).

### Biofilm analysis by scanning electron microscopy (SEM)

The anti-biofilm efficacy of NM was also assessed using SEM, where human nails (kindly provided by Dr Melyssa Negri, UEM, Paraná) were used as substrate. The procedure was approved by the Ethical Committee on Human Experimentation, under the number 31,702,520.40000.0104. The nail fragments were donated by healthy adult women. All nails used in these experiments were cut into fragments measuring 4–6 mm and meticulously cleaned using lancets to remove any dirt. Before the experiments, the fragments were sterilized by immersing them in 92% ethanol for at least 3 h and then washed 3× with phosphate-buffered saline (PBS). Then, *C. auris* cells (2 × 10^6^ cells/mL) in 500 µL of RPMI were incubated with the nail fragments at 37°C for 24 and 48 h. Following the incubation period, the cells were washed with PBS and treated with 25 μg/mL of NM in 500 µL of RPMI for 24 h. Subsequently, the nail fragments underwent PBS wash, and the adherent fungal cells and nails were fixed using a solution of 2.5% glutaraldehyde and 4.0% formaldehyde in 0.1 M cacodylate buffer at pH 7.2 for 2 h at room temperature. Next, the nail fragments were rinsed with cacodylate buffer, dehydrated using an ethanol gradient, and dried with Hexamethyldisilazane (HMDS). A 20 nm layer of gold was applied to the samples mounted on metal stubs using the Sputter coater SCD050 (LEICA). The samples were visualized, and digital images were taken using a scanning electron microscope (ZEISS EVO MA 10, Germany) operating at 10 kV.

### Membrane permeability assay

A flow cytometry method was used to address membrane permeability after interaction with the compound. The fungi were seeded into a 24-wells plate (10^6^ cells/well) with 1 mL RPMI with MOPS (0.165 M) and either the vehicle (ethanol) or 0.7 µg/mL (MIC50) or 1.4 g/mL (MIC90) for CDC 384 and 1.4 µg/mL (MIC50) or 2.8 µg/mL (MIC90) for CDC 390 of the alkaloid for 24 h at 37°C. As a positive control of yeast death, the fungal cells were incubated for 30 min at a temperature of 100°C. Then, the cells were centrifuged at 2000 × *g* for 10 min at 4°C. Labeling was done with 100 µg/mL of propidium iodide (PI; Sigma – USA) for 15 min at 37°C, then the cells were washed 3× with PBS and taken for analysis. Data were acquired using a BD LSR Fortessa™ Cell Analyzer flow cytometer (BD Biosciences, San Diego, CA, USA) and analyzed using FlowJo software (BD Biosciences).

### Alkaloid cytotoxicity in human fibroblasts

Alkaloid cytotoxicity was determined by the colorimetric MTT (3-(4, 5-Dimethylthiazol-2-yl)-2,5-Diphenyltetrazolium Bromide; Sigma – USA) assay using human dermal fibroblasts (HFb) (ATCC CRL-PCS-201-401). Cells were cultivated in RPMI containing 10% fetal bovine serum (FBS; Gibco – Brazil) and 1% penicillin-streptomycin (Gibco – USA). A final number of 2 × 10^5^ cells were plated onto 96-well plates in RPMI-FBS and then incubated (or not) with the NM or SM at concentrations ranging from 10, 25, 50, and 100 μg/mL. The plates were incubated at 37°C and 5% CO_2_ for 24 h. The supernatant was discarded, and the MTT solution (5 mg/mL) was diluted 1:10 in PBS and added to the plates. The plates were incubated with the MTT for 4 h at 37°C and 5% CO_2_. The blue formazan crystals were then solubilized with dimethyl sulfoxide (DMSO; Sigma – USA) and the solution absorbance was quantified using a microplate reader at 540 nm (Biotek ELx808™). Percentage of cytotoxicity was calculated through the following equation: % Cytotoxicity = 100 * (OD_570_ control group – OD_570_ treated group)/OD_570_ control group. All the assays were performed in quintuplicates.

### Selective index

The relationship between cytotoxicity and antifungal activity was determined using the Selectivity Index (SI), which was calculated by the ratio of the cytotoxic concentration 50% (CC_50_) (concentration required to reduce host cell viability by 50%), and the MIC value for strains (SI = [CC50]/[MIC]). The CC_50_ was determined using GraphPad Prism 8 (GraphPad, California, USA).

### In vivo toxicity of alkaloids

Larvae of *Galleria mellonella* were used to evaluate the cytotoxicity of the alkaloids [59]. Groups of 15 animals (weighing 0.2–0.3 g) were injected with 10 µL of alkaloids (100, 50, 25, or 10 µg/mL) into the left foremost proleg using a Hamilton micro-syringe. A ranging mass per animal of 5, 2.5, 1.25, and 0.5 µg of alkaloids was tested. PBS and ethanol (used to solubilize the alkaloids) were also injected as controls. After inoculation, larvae were placed in sterile Petri dishes and incubated at 37°C. Survival was recorded daily during a period of 7 days. The larvae were considered dead when no movement was apparent upon physical stimulation. Larval tegument melanization, an indicative of physical stress, was an additional factor observed.

### Galleria mellonella infection model

To test the effects of alkaloids *in vivo,* we employed a previously established model [59] with minor modifications. Briefly, groups of 15 larvae of *G. mellonella* were inoculated with *C. auris* strains CDC 384 or 390 (4 × 10^5^ cells in 10 μL) and incubated at 37°C. After 24 h, larvae were injected with 10 µL of 0.5, 5 50 µg/mL of NM or SM per animal. Control larvae were injected with either PBS or AMB. After injection, the larvae were placed in sterile Petri dishes and incubated at 37°C. Survival was recorded daily over a period of 7 days. The larvae were considered dead when no movement was apparent upon physical stimulation, as above.

### Statistics

All experiments were performed in triplicates or quintuplicates, in two or three independent experimental sets. The results were analyzed statistically by one-way analysis of variance (ANOVA). The correlation tests were determined by Dunnett’s correction. Survival analysis by log-rank (Mantel-Cox) test. All analyses were performed using the software GraphPad Prism5. In all analyses, *p*-values of 0.05 or less were considered statistically significant.

### Data availability

The data that support the findings of this study are openly available in Mendeley Data under the https://doi.org/10.6084/m9.figshare.24261106.v1.

## Results

### Natural solenopsins extract

A GC-MS chromatogram illustrating the composition of the NM extract is shown in Fig. S1, which displays six well-defined peaks corresponding to the following identified alkaloids: (1) isosolenopsin A, (2) dehydrosolenopsin B, (3) solenopsin B, (4) dehydrosolenopsin C, (5) solenopsin C, and (6) solenopsin D. The final concentrated extract is an oily pale-yellow liquid. The composition of the extract matched the same compounds and approximate relative proportions of alkaloids in *S. invicta* workers’ venom described by Liu et al. [45]. The GC-MS chromatogram for SM illustrated the relative proportions of four peaks corresponding to the main synthetic analogues for dehydrosolenopsin B, solenopsin B, dehydrosolenopsin C, and solenopsin C, at the exact retention times as the natural compounds. The SM, mimicking NM, appeared similarly oily, though slightly more translucid; both mixtures maintained the same aspect throughout the experiments.

### Solenopsins display a potent antifungal activity

The ability of solenopsins to inhibit fungal growth was tested against six strains of *C. auris*. Independent of the strain, both NM and SM showed potent inhibition of fungal growth at low concentrations (Table 1). Similar inhibitory concentrations were observed for both NM and SM. The IC_50_ against *C. auris* strains CDC 381, CDC 383, CDC 384 and CDC 385 was slightly lower (0.7 µg/mL) than that determined for strains CDC 382 and CDC 390 (1.4 µg/mL), which is one dilution different. Our results confirmed that both *C. auris* strains were resistant to FLU and that strain CDC 390 was also resistant to AMB (data not shown) [60].

**Table 1.** *In vitro* activity of the piperidine alkaloid solenopsins (NM: a natural extract from fire ants; SM: a mixture of synthetic analogues) against the yeast *Candida auris*.

	IC_50_ (µg/mL)	
	NM	SM*
*C. auris* strain		
CDC 381	0.7	0.7
CDC 382	1.4	1.4
CDC 383	0.7	0.7
CDC 384	0.7	0.7
CDC 385	0.7	0.7
CDC 390	1.4	1.4

IC_50_: concentration of alkaloids required to inhibit *C. auris* growth by 50%.

*Synthetic mixture containing dehydrosolenopsin B, solenopsin B, dehydrosolenopsin C, and solenopsin C.

### Additive effect between alkaloids and amphotericin B

Given the potent activity of the alkaloids (NM and SM) against various *C. auris* strains, we sought to determine whether combining them with AMB would enhance the antifungal activity or restore efficacy against resistant strains of *C. auris*. Since both NM and SM displayed the same IC_50_ values, we prioritized NM due to its higher availability. We tested two different strains (CDC 384 and CDC 390) to assess whether the intrinsic resistance against FLU (CDC 384 and CDC 390) and AMB (CDC 390) would be impacted by the alkaloids. As seen in Table 2, indifferent interactions (i.e. FICi ˃0.5–4.0) were observed with the alkaloid-AMB combination in both *C. auris* strains tested using the checkerboard method. However, when synergism was analyzed following the BLISS model, which assumes that the drugs act independently, the natural alkaloids and AMB showed additive interaction when tested against *C. auris* CDC 384 and *C. auris* CDC 390. Comparatively, by checkerboard and BLISS method, the alkaloid-FLU combination delivered indifferent effect on both *C. auris* strains tested (data not shown). Hence, the inhibitory effect of the combination was generated by the alkaloids, with no variation in the FLU MIC.

**Table 2.** *In vitro* combined activities of a natural extract of the piperidine alkaloid solenopsins (NM) and the antibiotic amphotericin B against strains of the yeast *Candida auris*.

*Candida* spp. /Drug combination	Checkerboard test		Bliss model	
	Ʃ FIC	Activity	Score	Activity
*C. auris* CDC 384 NM – Amphotericin B	0.59	Indifferent	1.58	Additive
*C. auris* CDC 390 NM – Amphotericin B	1.03	Indifferent	3.03	Additive

### Solenopsins alkaloids impact on *C. auris* biofilm

**Figure 1.**
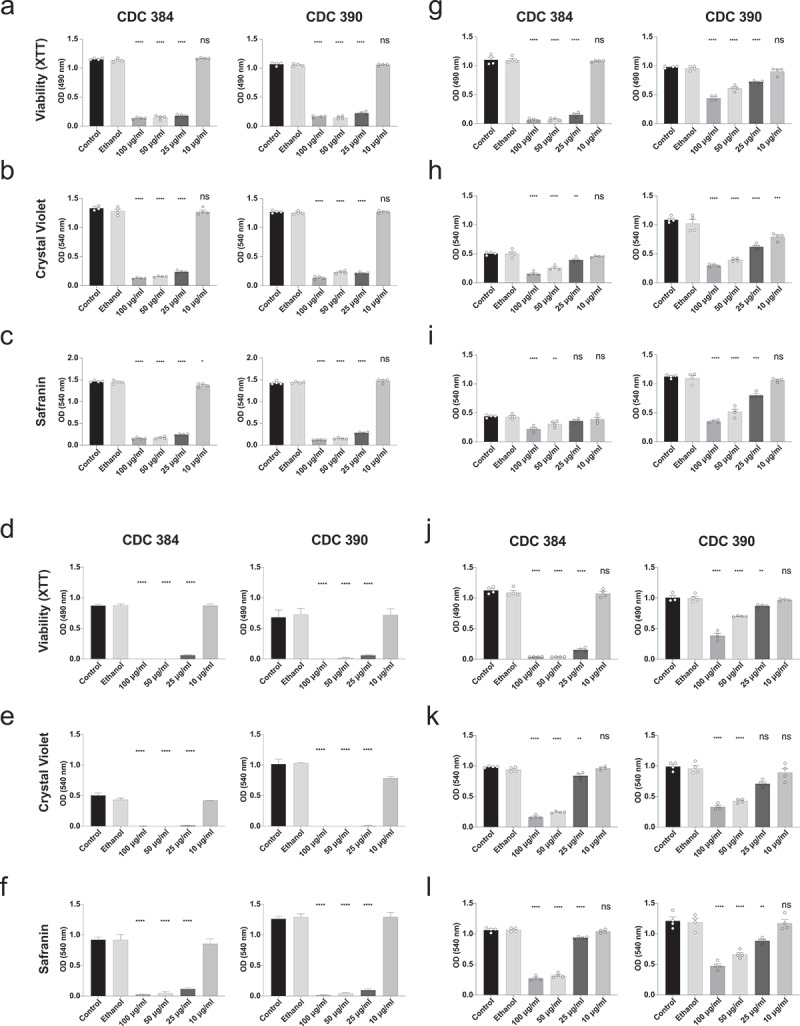
Effect of piperidine alkaloids solenopsins (NM: a natural fire ant extract; SM: a synthetic mixture of analogues) on biofilm formation by the yeast *Candida auris*. (a–f) Biofilm formation was evaluated after 48 h of growth. *C. auris* yeast strains CDC 384 or 390 (each at 10^6^ cells/200 µl) were incubated for 90 min, the nonadherent cells were removed by washing with PBS, fresh medium containing different concentrations of NM or SM, and the cells were cultivated for 48 h at 37°C. (g–l) Alternatively, the effect of NM or SM was also evaluated on mature biofilm. Biofilms formed after 24 h were treated with different concentrations of NM or SM for additional 24 h. Metabolic activity (XTT) and biomass (crystal violet and safranin stainings). The results are shown as mean ± standard deviation from three independent experiments. Group comparisons were performed with one-way analysis of variance (ANOVA) with Dunnett’s correction (** *p* = 0.01, **** *p* < 0.0001) compared to the ethanol group.

To investigate the effect of the alkaloids on *C. auris* biofilm, we conducted experiments using two strains (CDC 384 and CDC 390) under different conditions. Initially, the alkaloids were introduced after *C. auris* cells had attached to the plate. In control conditions, where alkaloids were absent, both strains formed biofilms. However, as depicted in Figure 1a–f, SM and NM exhibited strong inhibitory effects on biofilm formation in both strains. They markedly reduced metabolic activity and biomass across all concentrations tested, except for the lowest concentration (10 µg/mL) (Figure 1a-f). Notably, SM appeared to be more effective in this context. Furthermore, treatment with either NM or SM significantly reduced the metabolic activity of mature biofilms (Figure 1g–l). The mature biofilm formed by the CDC 390 strain showed less susceptibility to NM treatment. Additionally, NM’s impact on biomass was less pronounced compared to its effect on biofilm formation in both strains. Under these conditions (treatment of mature biofilm), NM and SM exhibited comparable activities. The ethanol concentration used in our assays had no impact on the biofilm properties.

**Figure 2.**
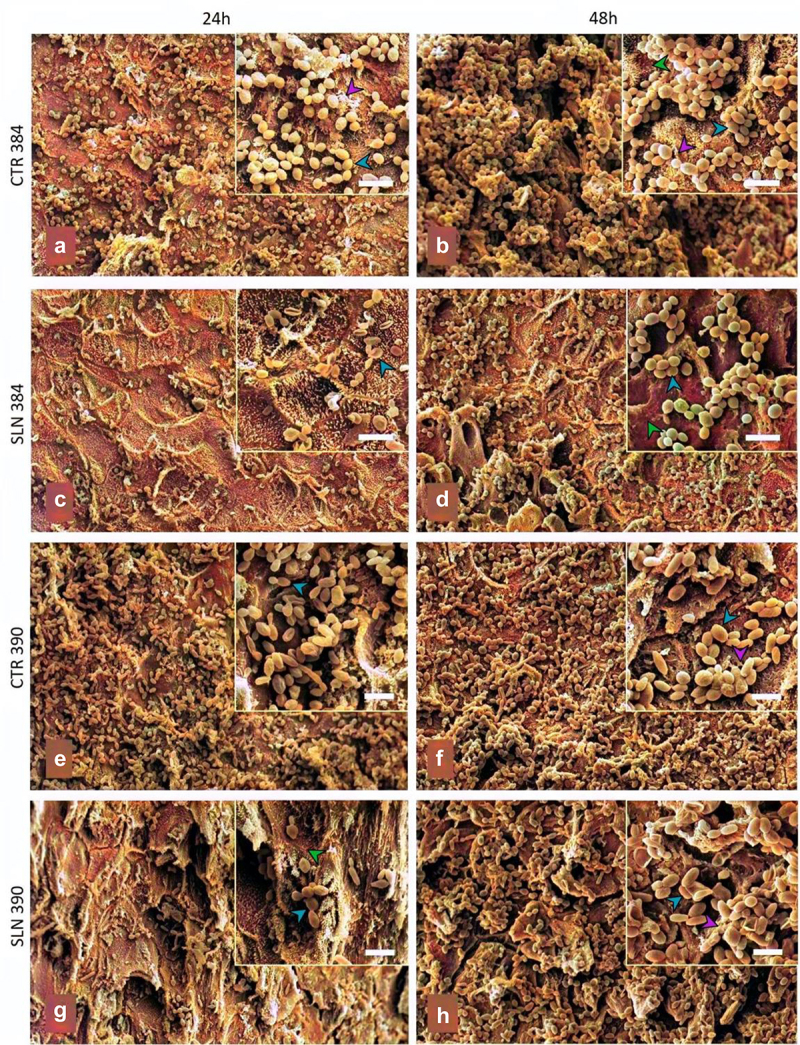
Effect of the natural extract (NM) on *Candida auris* biofilm formation on human nail fragments. *C. auris* strains (CDC 384 or CDC 390) were cultured in SDB in the presence of human nail fragments, followed by incubation at 37°C for 24 (a, c, e, and g) or 48 h (b, d, f, h). Post-cultivation, the nail fragments underwent triple washing with PBS and were subsequently treated with 25 µg/mL of NM for 24 h before processing for scanning electron microscopy (SEM). Insets provide higher magnification, with a scale bar of 10 µm. In the insets, a blue arrowhead points to a yeast, while green and purple arrowheads indicate the biofilm matrix and the nail surface, respectively.

SEM analysis confirmed these findings, demonstrating a considerable reduction in yeast numbers following NM treatment for both formation and mature biofilm conditions (Figure 2). Notably, while NM treatment was highly effective against the CDC 390 strain during biofilm formation, a more persistent biofilm was observed when treating mature biofilms, indicating increased resistance of this strain to NM under these conditions.

### Solenopsin alkaloids promote C. auris membrane disruption

**Figure 3.**
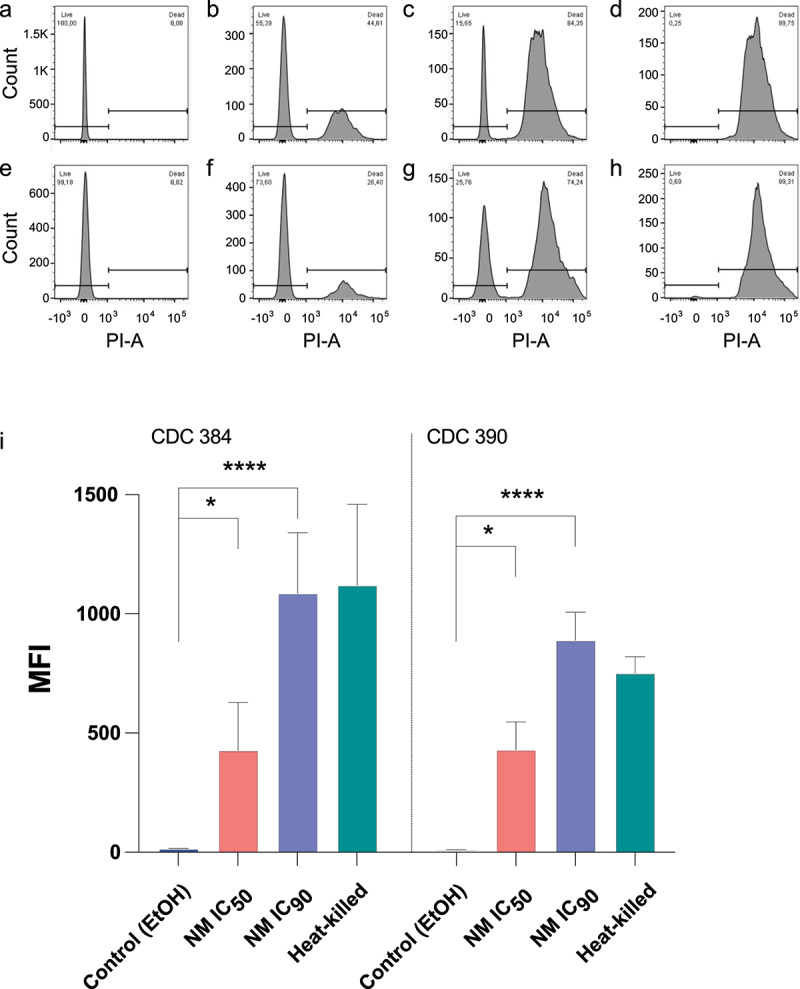
Cell viability of yeast *Candida auris* strains exposed to piperidine solenopsin alkaloids. (a-h) *C. auris* yeast cells (CDC 384 and CDC 390 strains) were treated with ethanol (1% in PBS) (EtOH) or NM (IC_50_ or IC_90_) for 24 h and then stained with propidium iodide (PI) to determine cell viability (membrane permeability). IC_50_ and IC_90_ represent the concentrations of NM capable of inhibiting 50% and 90% of growth, respectively. Heat-killed cells were used as a control. After treatment, yeast cells were analyzed by flow cytometry. Results are representative of three independent experiments with similar results. (i) Fluorescence intensity of pi-labelled cells in each treatment condition was compared. Group comparisons were performed with one-way analysis of variance (ANOVA) with Dunnett’s correction (* *p* = 0.03, **** *p* < 0.0001) compared to the ethanol-treated group.

To explore the mechanism of fungicidal activity displayed by the alkaloids, we assessed cellular membrane disruption using propidium iodide (PI) after treating the fungi with two concentrations of NM (MIC_50_ and MIC_90_) for 24 h. Exposure to these alkaloids culminated with membrane disruption-mediated cell death in a dose-dependent fashion (Figure 3). *C. albicans* strain CDC 384 displayed cell death of 44% and 84% after treatment with NM at MIC_50_ and MIC_90_, respectively (Figure 3b,c). Strain CDC 390 was slightly more resistant, showing death of 26% and 74% after treatment with the same alkaloid extract concentrations (Figure 3f,g). Heat-killed cells were used as a positive control (Figure 3d,h).

### Toxicity of alkaloids to mammalian cells and Galleria mellonella larvae

**Figure 4.**
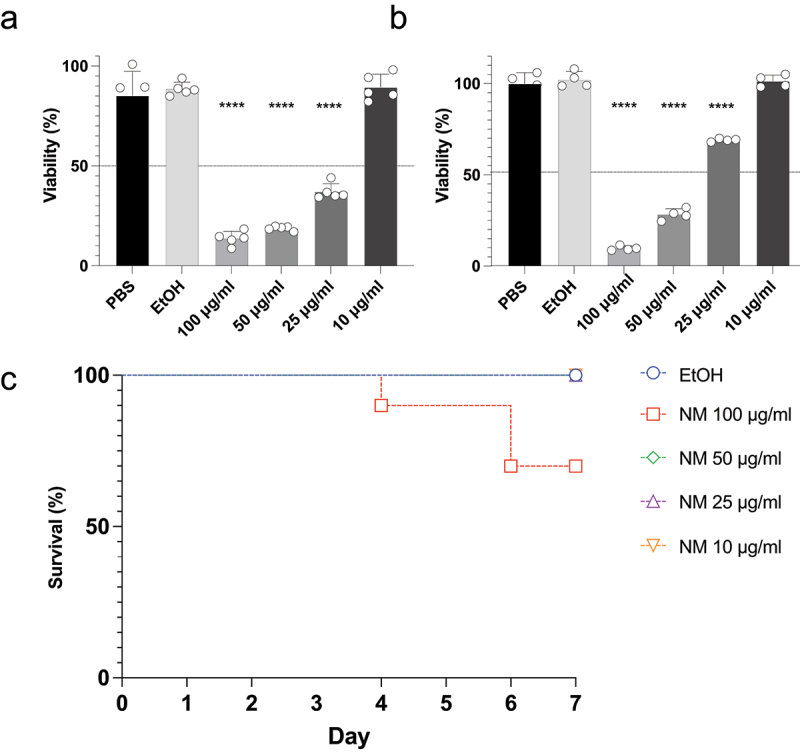
Toxicity of the solenopsin alkaloids against human fibroblasts and insect larvae of *Galleria mellonella*. Human dermal fibroblasts (2 × 10^5^ cells per well) were treated with a natural extract of solenopsins NM (a) or a synthetic mixture of syntetic analogues SM (b) at 10, 25, 50, and 100 µg/mL, and their viability determined using MTT. Controls were performed using PBS or ethanol (EtOH) (1% in PBS). (c) Larvae from *G. mellonella* were injected with NM (10, 25, 50, and 100 µg/mL) and mortality was monitored for 7 days. Ethanol (EtOH) (1% in PBS) was used as control. No larval death was detected when NM was used at concentrations of 10, 25, and 50 μg/mL, as well as with the control, EtOH. Data (in a and b) were analyzed by one-way analysis of variance (ANOVA) followed by Dunnett’s correction (**** = *p* < 0.0001) and the difference between groups (in c) was analyzed by log-rank (Mantel-Cox) test (* = *p* < 0.05).

We investigated the toxicity of solenopsins to human dermal fibroblasts. Vehicle (1% ethanol in PBS) treatment did not change the viability of the fibroblasts (Figure 4a). Treatment with NM or SM in concentrations above 25 µg/mL significantly decreased cell viability in a dose-dependent fashion, but no significant decrease in viability was observed at 10 µg/mL.

We also investigated the effect of NM *in vivo*, using larvae from *G. mellonella* as a model. Remarkably, no toxicity as measured by survival was observed when the larvae were injected with 50, 25, or 10 µg/mL of a natural solenopsins per animal (Figure 4b). At 100 µg/mL, 72% of the larvae survived treatment.

### Selective Index (SI) of NM and SM

The selectivity index (SI) is commonly used to assess the cell selectivity of an antimicrobial therapeutic compound. Based on our data, the CC50 values for NM and SM, representing the concentrations required to reduce cell viability by 50%, are 37.66 and 40.22, respectively (Table 3). Subsequently, we calculated the SI for both alkaloid mixtures, and the values exceeded 10 for both mixtures against all tested strains (Table 3). This indicates a high level of selectivity against the fungus.

**Table 3.** Selective Index (SI) for natural and synthetic mixtures (NM and SM).

Compound	Strains	IC _50_ (µg/ml)	CC _50_ (g/ml)	Selectivity Index (SI)
NM	CDC 381, 383, 384 and 385	1.4	37.66	26.9
	CDC 382	2.8		13.4
SM	CDC 381, 383, 384 and 385	1.4	40.22	28.7
	CDC 382	2.8		14.3

IC50: concentration of alkaloids required to inhibit C. auris growth by 50%.

CC50: concentration of alkaloids required to reduce cell viability of host cells (fibroblasts) by 50%.

### Solenopsins showed a potent effect against candidiasis *in vivo*

**Figure 5.**
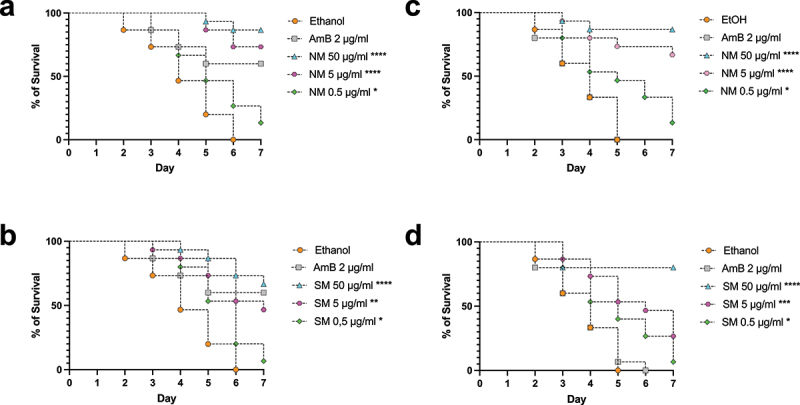
The piperidine alkaloids solenopsins control candidiasis in insect larvae of *Galleria mellonella*. Lethal suspensions of *Candida auris* yeast cells (strain CDC 390: a and b; strain CDC 384 c and d) were injected into *G. mellonella* larvae (4 × 10^5^ cells per insect). After 24 h, different doses of either a natural mixture (NM) or a synthetic mixture (SM) (0.5, 5, 50 µg/ml) were administered to the larvae. Amphotericin B (2 µg/ml) was used as a positive control. The difference between vehicle treatment and each group was analyzed by log-rank (Mantel-Cox). * = *p* < 0.05, ** = *p* < 0.01, *** = *p* < 0.001, and **** = *p* < 0.0001.

We investigated whether treatment with NM or SM would control infection of *G. mellonella* with *C. auris*. The larvae were challenged with lethal amounts of yeasts, and non-toxic concentrations of NM or SM were injected 24 h later. Untreated larvae died up to 6 days after infection (Figure 5). The alkaloids displayed a dose-dependent protection of the larvae from a lethal challenge with both strains of *C. auris* (CDC 384 and CDC 390) (Figure 5). The lowest dose of alkaloids (0.5 µg/mL) conferred a small but significant protective effect. Remarkably, 5 µg/mL of NM conferred a protective effect similar in potency to that observed with AMB for strain CDC 384. The highest dose of alkaloids (50 µg/mL) was more effective in protecting the larvae than was the AMB for the same strain. NM showed a tendency to be more effective to protect the insects. Inoculation of vehicle (ethanol) alone, in the absence of an infection, did not have any effect on the survival of the larvae.

## Discussion

Piperidine alkaloids have historically been known as bioactive secondary metabolites of plants, frequently studied and exploited for their antimicrobial effects, particularly as fungicides [61]. The fire ants were the very first animals discovered to produce alkaloid-rich venoms, containing piperidines [62]. Various classes of plant alkaloids have demonstrated the ability to control fungal growth. However, the activities of solenopsins have been less extensively investigated, as a unique set of piperidine alkaloids derived from animals. Their fungicidal activity has been demonstrated mainly with fungi of ecological importance [39,40], such as plant- or entomo-pathogens (reviewed in [36]). Therefore, there is a clear gap in the literature regarding the activity of solenopsins against human-pathogenic fungi [33,34].

The NM fire ant extract tested in this study contained a complex mixture of 10 solenopsin analogs (i.e. six main identified plus 4 minor compounds), corresponding to what was demonstrated in previous studies [45,47]. The predominant compounds were *trans*- isomers of saturated and unsaturated analogs of solenopsins B and C. Across most experiments, the NM activity was comparable with that of the SM, which contained synthetic analogues of the predominant alkaloids in the same approximate relative proportions observed in the NM. Previous studies demonstrated that these alkaloids display antibacterial, antiprotozoan and antifungal properties [37,63,64]. Taken together, to date fire ant solenopsins have consistently demonstrated pronounced effects against fungi of biomedical, entomopathogenic, and agricultural interest [40,41,62].

The antifungal effect of NM or SM on *C. auris* planktonic cells was comparable in potency to the effect of AMB. The alkaloids were also effective against the *C. auris* strains CDC 384 and CDC 390, which are resistant to FLU alone and both FLU and AMB, respectively, suggesting that these compounds can be potential alternatives to overcome resistance in fungi. In addition, our data also indicate that these alkaloids can be administered in combination with AMB, resulting in an additive effect against the *C. auris* strains. Since these alkaloids are active against strains of other *Candida* species, such as *C. albicans*, *C. glabrata*, and *C. krusei,* as well as against *Cryptococcus neoformans* [65,66], they have a broad effect for human fungal pathogens and can be further exploited as a new strategy for combatting fungal disease.

Antifungals become less effective when dealing with yeast cells within a biofilm [67]. Although higher concentrations of solenopsins were required, the solenopsins inhibited, in a dose-dependent fashion, biomass accumulation within *C. auris* biofilms. This effect could enhance the delivery of the antifungals to the yeast cells [67], which may also explain the impact of the solenopsins on the metabolic activity of yeast cells in the biofilm. Apart from direct antimicrobial effects attributed to solenopsins, an increased reactive oxygen species (ROS) in eukaryotic cells exposed to synthetic solenopsin A and other synthetic analogs has been reported [68], which seems to be at least partially responsible for the solenopsin mechanism of action onto *C. auris*. Indeed, the antimicrobial mechanism of action of solenopsins has remained a controversial topic [31], with some authors suggesting the amphipathic nature of these molecules could interfere with cellular membranes [69] and others claiming their toxicity derived from their longest alkyl-side chains [65]. Our data show that the treatment with solenopsins promotes membrane disruption of *C. auris* yeast cells, suggesting that the inability of treated yeasts to maintain membrane integrity could be part of the mechanism of action of solenopsin alkaloids. Other mechanisms could inhibit fungal growth, for instance, these compounds have been demonstrated to suppress quorum-sensing signaling, modulating virulence factors in the pathogenic bacterium *Pseudomonas aeruginosa* [38], but this would require further experimentation to assess in *C. auris*.

As well as other antifungal compounds, alkaloids can display some level of toxicity to the host [36]. However, both NM and SM displayed a high selective index (SI) (>10), which indicates a considerable selectivity of the alkaloids to the yeasts over the mammalian cells. In fact, other piperidine derivatives have been extensively investigated and are currently used in humans for pain relief, anesthesia, and treating psychiatric disorders [70]. In addition, piperidine derivatives represent one of the most important synthetic medicinal blocks for drug construction, which make solenopsin alkaloids as potential scaffolds for more active and less toxic compounds to combat fungal infections.

We further addressed the toxicity of solenopsins *in vivo* using a *Galleria mellonella* model. The larvae of *G. mellonella* are considered a classical model for studying toxicity for being large, easy to rear and manipulate [71]. In addition, the therapeutic doses of antibiotics used in *G. mellonella* may displayed a good correlation with human treatments. The insect larvae displayed significant resistance to the alkaloids (Figure 3b), being only slightly affected by the highest dose of the solenopsins extract. Piperidine alkaloids are classically known and studied as secondary metabolites of plants, with the fire ants being one of the few animal organisms producing such compounds [33]. Therefore, it is possible that *G. mellonella* larvae exhibit particular resistance to solenopsins, suggesting a limitation of the model that had not been examined.

To evaluate if there is an effective therapeutic window for the treatment of candidiasis with these alkaloids, larvae of *G. mellonella* were treated with solenopsins 24 h after infection with *C. auris*. Corroborating the obtained SI values, the solenopsins were highly protective of the larvae across all tested concentrations, suggesting that there is a significant distance between toxic and therapeutic concentration dosages, allowing for effective treatment. As observed previously for the *in* vitro studies, the alkaloids were efficient against the strains resistant to FLU and AMB, confirming that they may work as an alternative in cases of resistance against the classical antifungal drugs. Similar results were previously shown for piperine alkaloids used against *C. albicans* in other models [72,73]. Interestingly, the use of the synthetic mixture of solenopsins closely replicated the protective effect of the natural extract in protecting *G. mellonella* from candidiasis by *C. auris*. This confirmation establishes that piperidine alkaloids, like solenopsins, could be further explored as potential therapeutical tools to be used either alone or in combination with classic antifungals in the treatment of candidiasis.

## Supplementary Material

Figure_S1.docx
