# Supplementary material for: Alkaloids solenopsins from fire ants display in vitro and in vivo activity against the yeast Candida auris
Source: Virulence. 2024 Oct 7;15(1):2413329. doi: 10.1080/21505594.2024.2413329 (PMC11469440; doi:10.1080/21505594.2024.2413329)
Supplement: Figure_S1.docx [file KVIR_A_2413329_SM4509.docx]

Figure S1. Gas-chromatography coupled with Mass Spectrometry (GC-MS)
of main piperidine venom alkaloids in the venom of Solenopsis fire
ants. (A) venom extract of Solenopsis invicta from Guangzhou, China;
(B) synthetic mixture mimicking the natural extract above. Numbers
correspond to compounds identified by their mass spectra (Chen &
Fadamiro 2009): (1) isosolenopsin A; (2) Dehydrosolenopsin B; (3)
Solenopsin B; (4) Dehydrosolenopsin C; (5) Solenopsin C.
